# LINC02159 promotes non-small cell lung cancer progression via ALYREF/YAP1 signaling

**DOI:** 10.1186/s12943-023-01814-x

**Published:** 2023-08-04

**Authors:** Qiurong Yang, Maoye Wang, Jing Xu, Dan Yu, Yixin Li, Yanke Chen, Xiaoxin Zhang, Jiahui Zhang, Jianmei Gu, Xu Zhang

**Affiliations:** 1 Department of Laboratory Medicine, School of Medicine, Jiangsu University, Zhenjiang, 212013 China; 2 Departmemt of Clinical Laboratory Medicine, Nantong Tumor Hospital/Affiliated Tumor Hospital of Nantong University, Nantong, 226300 China

**Keywords:** LncRNA, NSCLC, ALYREF, YAP1, m^5^C modification, Progression

## Abstract

Lung cancer is the leading cause of cancer-related deaths worldwide. Long non-coding RNAs (lncRNAs) have emerged as key regulators of cancer development and progression, and as promising biomarkers for the diagnosis and prognosis of cancer. In this study, we identified a new lncRNA (LINC02159) that was upregulated in the tumor tissues and serum of non-small cell lung cancer (NSCLC) patients. We demonstrated that knockdown of LINC02159 inhibited NSCLC cell proliferation, migration, and invasion, but induced cell apoptosis and cell cycle arrest in vitro and retarded tumor growth in vivo, while overexpression of LINC02159 led to the opposite effect. We discovered that LINC02159 was highly correlated with cancer growth and metastasis-related pathways by using transcriptomic analysis and that YAP1 was a potential target gene of LINC02159. Mechanistically, LINC02159 bound to the Aly/REF export factor (ALYREF) to enhance the stability of YAP1 messenger RNA (mRNA) via m^5^C modification, which led to the overexpression of YAP1 and the activation of the Hippo and β-catenin signaling pathways in NSCLC cells. Rescue experiments showed that LINC01259 promoted NSCLC progression in a YAP1- and ALYREF-dependent manner. In conclusion, LINC02159 plays an oncogenic role in NSCLC progression by regulating ALYREF/YAP1 signaling, and it has the potential to be utilized as a diagnostic marker and therapeutic target for NSCLC.

**Supplementary Information:**

The online version contains supplementary material available at 10.1186/s12943-023-01814-x.

## Introduction

Lung cancer is the main cause of cancer-related death worldwide [1]. Non-small cell lung cancer (NSCLC) accounts for about 85% of all lung cancer cases [2]. Although there have been tremendous advancements in detection and treatment in the past decade, the early diagnosis rate, overall cure rate, and survival rate of NSCLC remain low, especially in metastatic disease. Therefore, there is an urgent need to understand the molecular mechanism for NSCLC development and progression and to find more effective biomarkers and therapeutic targets.

Long non-coding RNAs (lncRNAs) are a highly heterogeneous group of transcripts consisting of various RNA species > 200 nucleotides in length, lacking apparent protein-coding potential, and regulating gene expression through multiple mechanisms [3]. In recent years, lncRNAs have been recognized as key players in human health and diseases. A growing body of evidence suggests that lncRNAs play a crucial role in the transcriptional and post-transcriptional regulation of gene expression. In terms of transcriptional regulation, lncRNAs in the nucleus control the epigenetic activation or silencing of gene expression [4, 5]. Meanwhile, lncRNAs in the cytoplasm are involved in post-transcriptional gene regulation. LncRNAs can affect the stability of mRNA as well as the translation and subcellular localization of target mRNA [6, 7]. The dysregulation of lncRNAs is closely related to the occurrence, development, metastasis, and drug resistance of cancers [8]. More importantly, the aberrant expression of lncRNAs in cancer has been considered a potential biomarker for cancer diagnosis and prognosis, and has been developed as potential targets for cancer therapy.

LncRNAs have been implicated as key players in NSCLC development and progression. For example, lncRNA CASC15 is highly expressed in NSCLC tissues and its upregulation is closely related to poor prognosis. CASC15 promotes NSCLC cell proliferation and migration through the HIF-1α/CASC15/SOX4/β-catenin regulatory axis [9]. In addition, lncRNA HOXC-AS3 promotes the growth and metastasis of NSCLC cells by stabilizing YBX1 and inhibiting its ubiquitination mediated by MDM2 [10]. Epidermal growth factor receptor (EGFR)-tyrosine kinase inhibitors (TKIs) are effective targeted therapies for patients with advanced NSCLC carrying sensitizing EGFR mutations. Studies have shown that lncRNAs LCETRL3 and LCETRL4 on chromosome 4q12 reduce the therapeutic sensitivity of EGFR-TKI in NSCLC by stabilizing TDP43 and EIF2S1 [11]. Ma et al. have constructed an immunotherapeutic response and immune-related (ITIR)-lncRNA signature, which can be used to predict the immunotherapy response and prognosis of NSCLC patients receiving immunotherapy [12]. Ling et al. demonstrated that the level of exosomal lncRNA RP5-977B1 has high diagnostic ability and prognostic value in NSCLC because it is higher than that in the healthy control group [13]. These findings indicate that lncRNAs are critically involved in the pathogenesis of NSCLC and can be used as biomarkers for the diagnosis and prognosis of NSCLC.

In this study, we have identified a new lncRNA, LINC02159, using RNA sequencing of paired tumor tissues and adjacent non-tumor tissues from NSCLC patients. We found that LINC02159 exerted oncogenic roles in NSCLC progression by using gain- and loss-of-function studies. Our proteomic and transcriptomic analysis results uncovered that LINC02159 bound to Aly/REF export factor (ALYREF) and enhanced the 5-methylcytosine (m^5^C) modification of YAP1 mRNA by ALYREF, which upregulated YAP1 expression and activated Hippo and β-catenin pathways in NSCLC cells. Our work identifies a new LINC02159/ ALYREF/YAP1 axis in NSCLC progression, and this may be a potential biomarker and therapeutic target for human NSCLC.

## Materials & methods

### Patients and clinical specimens

A total of 50 paired tumor and adjacent non-tumor tissues from NSCLC patients, 44 serum samples from NSCLC patients, 33 serum samples from pneumonia patients and 44 serum samples from healthy donors were obtained from Nantong Tumor Hospital between November 2018 and November 2019. None of the enrolled patients received chemotherapy, radiotherapy, immunotherapy, or other adjuvant therapy before surgery. Samples were collected in accordance with the institutional protocol. Immediately after collection, tissue samples were rapidly frozen in liquid nitrogen and stored at − 80℃ for further use. All the participants signed informed consent, and the study was approved by the Institutional Ethical Committee of Jiangsu University.

### Cell culture

Human NSCLC lines (A549, H1299, and PC9) and bronchial epithelial cells (HBE) were purchased from the Cell Bank of the Chinese Academy of Sciences (Shanghai, China). HBE, H1299 and PC9 cells were cultured in RPMI 1640 medium (Gibco, USA) with 10% fetal bovine serum (ExCell, USA) and 1% penicillin and streptomycin (Biosharp, China). A549 cells were cultured in DMEM/F12 medium (Meilunbio, China) containing 10% fetal bovine serum (ExCell) and 1% penicillin and streptomycin (Biosharp). All the cells were cultured at 37℃ in a humidified atmosphere with 5% CO_2_. All the cells used in this study were tested negative for mycoplasma.

### Plasmid and small interfering RNA (siRNA) transfection

Specifically targeted siRNAs and overexpression plasmids were designed and synthesized by GenePharma (Suzhou, China) and Hanbio (Shanghai, China). Cells were seeded in 6-well plates with a cell density of 2 × 10^5^/well and cultured overnight until the cell density was 50–70% confluent. According to the manufacturer’s instructions, siRNAs and plasmids were transfected into NSCLC cells using Lipofectamine 2000 (Invitrogen, USA) in a serum-free medium. Cells were changed to complete medium at 6 h after transfection and cultured for another 30 h. The sequences of siRNAs are summarized in Additional file1: Table S1.

### RNA fluorescence in situ hybridization (FISH)

For the FISH assay, an RNA FISH kit (GenePharma) was used according to the protocol, and the Cy3-labeled LINC02159 RNA probe was designed and synthesized by GenePharma. The cells were analyzed using super-resolution microscopy. The sequence of the RNA probe for LINC02159 FISH is listed in Additional file1: Table S1.

### Tagged RNA affinity purification (TRAP) assay

A TRAP Kit (TRAP, Bersinbio, China) was applied to detect the interaction between lncRNA and proteins. Control and LINC02159 overexpression vectors that contain MS2 stem-loop structure (MS2 and lncRNA-MS2) and GST-MS2 overexpression vector were designed by Bersinbio. Then, MS2 and lncRNA-MS2 vectors were co-transfected with GST-MS2 into NSCLC cells to obtain the complex of GST-MS2 ~ lncRNA-MS2 and interacting proteins. Finally, the transfected cells were lysed, and the complex was extracted by using glutathione affinity magnetic beads to obtain the target proteins. Mass spectrometry identified the lncRNA-binding proteins, and the TRAP experiment was verified by silver staining and western blot.

### LC–MS/MS

Protein samples digested with sequencing-grade trypsin were analyzed by liquid chromatography-tandem mass spectrometry (LC-MS/MS) to obtain original mass spectrometry results. A byonic software was used to analyze the raw file and search the uniprot-Homo sapiens data to obtain the identified protein results.

### RNA-sequencing analysis

Total RNA was extracted from control and LINC02159 knockdown NSCLC cells, and RNA sequencing was performed by OEBiotech (Shanghai, China). Differentially expressed genes were screened and identified mainly through the set |log2 (FoldChange)| and *p* values. Finally, differentially expressed genes were functionalized by Gene Set Enrichment Analysis (GSEA), Kyoto Encyclopedia of Genes and Genomes pathway (KEGG) analysis, and Protein-Protein Interaction Networks (PPI).

### RNA immunoprecipitation (RIP) assay

RIP assay was performed using an RIP kit (Genseed, China). Approximately 1 × 10^7^ cells were collected and lysed with RIP lysis buffer. Anti-ALYREF, anti-m^5^C, or control IgG (5 µg) were incubated with magnetic beads at 4 °C for 2 h. Cell lysates were then incubated with the magnetic beads at 4 °C overnight. Finally, the immunoprecipitated RNA complexes were purified and quantified by quantitative real-time PCR (qRT-PCR). Antibody information is listed in Additional file 1: Table S3.

### In vivo animal studies

For the xenograft tumor model, 4-week-old male BALB/c nude mice were purchased from the Model Animal Research Center of Nanjing University (Nanjing, China) and raised under specific pathogen-free (SPF) conditions according to the National Institutes of Health (NIH) guidelines for the care and use of mice. Mice were randomly divided into 2 groups (n = 5 for each group) and subcutaneously injected with A549 cells (5 × 10^6^ cells per mouse) that were transfected with LINC02159 siRNA or control vector. The tumor size was measured every 3 days, and the formula for calculating tumor volume was: volume = 0.5×length×width^2^. Tumor tissues were harvested for hematoxylin and eosin (H&E) and immunohistochemistry (IHC) staining. The animal study has been approved by Jiangsu University Animal Use and Care Committee.

### Statistical analysis

Statistical analysis was performed using SPSS software (Chicago, USA). Student’s t-test and χ^2^-test were carried out to analyze the comparison of differences between two groups and multiple groups. GraphPad Prism 7 software was used for plotting. *P* < 0.05 was considered statistically significant (*P < 0.05, **P < 0.01, ***P < 0.001).

## Results

### LINC02159 is upregulated in NSCLC and associated with poor prognosis

**Fig. 1:**
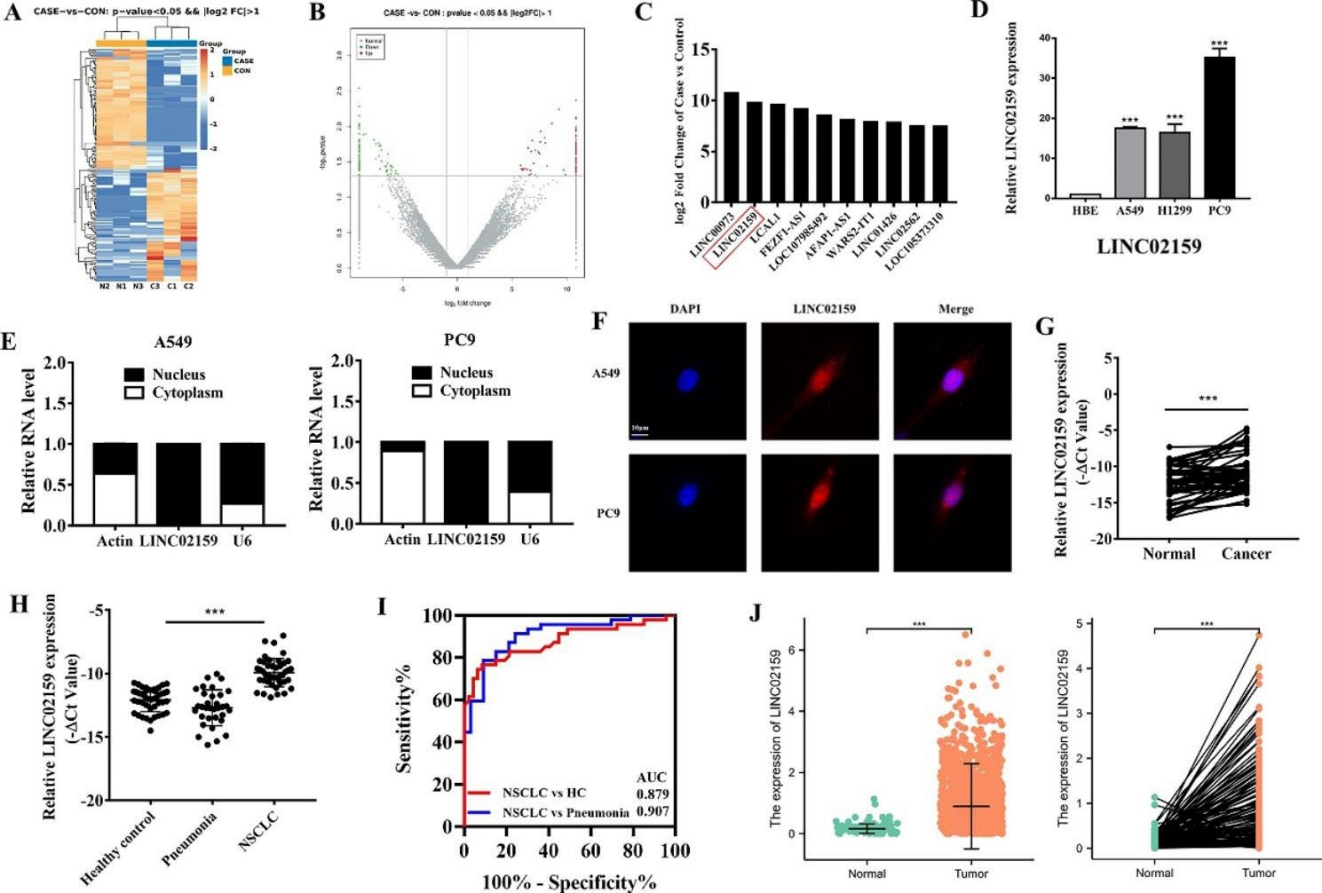
LINC02159 is significantly upregulated in NSCLC and associated with poor prognosis. **A** Cluster analysis of differential lncRNA expression in 3 pairs of NSCLC tissues and adjacent non-tumor tissues. **B** Volcano map of differential lncRNA expression. **C** Top ten lncRNAs with significant upregulation. **D** qRT-PCR analyses of LINC02159 expression in different lung cancer cell lines. **E** RNA nuclear/cytosol fractionation experiment. **F** FISH experiment for the subcellular distribution of LINC02159 in NSCLC cells. **G** qRT-PCR analyses of LINC02159 expression in 50 pairs of NSCLC tissues and adjacent non-tumor tissues. **H** qRT-PCR analysis of LINC02159 expression in the serum of NSCLC patients, pneumonia patients and healthy controls. **I** ROC curves among NSCLC patients, healthy controls, and pneumonia patients. **J** TCGA database analysis of LINC02159 expression in tumor and normal tissues of NSCLC patients. (***P < 0.001)

To identify the deregulated lncRNAs in NSCLC, we performed RNA sequencing by using matched tumor tissues and adjacent non-tumor tissues from NSCLC patients (n = 3). The results of RNA sequencing showed that there were 84 upregulated lncRNAs and 89 downregulated lncRNAs in tumor tissues compared to adjacent non-tumor tissues (Fig. 1A and B). The top ten upregulated lncRNAs in tumor tissues were listed in Fig. 1C, including several lncRNAs that have been previously reported to be critically involved in cancer progression, such as FEZF1-AS1 and AFAP1-AS1. Of these, we chose LINC02159 for further study as it has not been studied in cancer until now. We verified that LINC02159 expression was upregulated in human NSCLC cell lines (A549, H1299, and PC9) compared to HBE cells (Fig. 1D). We then examined the distribution of LINC02159 in NSCLC cells by nuclear/cytosol fractionation and FISH experiments, and found that LINC02159 was mainly localized in the nucleus of NSCLC cells (Fig. 1E and F).

We next detected the expression of LINC02159 in paired tumor tissues and adjacent non-tumor tissues from NSCLC patients (n = 50). qRT-PCR results showed that LINC02159 expression levels were higher in 72% of tumor tissues than in adjacent non-tumor tissues (Fig. 1G). Meanwhile, we observed that the expression of LINC02159 was significantly upregulated in the serum of NSCLC patients compared to those of pneumonia patients and healthy individuals (Fig. 1H). The area under the receiver operating characteristic curve (AUC) was 0.879 between NSCLC patients and healthy individuals, with a sensitivity of 76.6% and a specificity of 91.49%, while the AUC was 0.907 in differentiating NSCLC patients and pneumonia patients with 78.72% of sensitivity and 90.91% of specificity (Fig. 1I). Additionally, we also found that there was a positive correlation between LINC02159 and CEA levels (Figure S1). We further analyzed LINC02159 expression by using TCGA data and the results also showed an upregulation of LINC02159 in tumor tissues of NSCLC patients (Fig. 1I). The elevated expression of LINC02159 could be observed in tumor tissues of all-stage NSCLC patients (Figure S1). Moreover, those patients harboring a higher level of LINC02159 tended to have a shorter overall survival (OS) time (Figure S1). In summary, these data indicate that LINC02159 may be used as a biomarker for NSCLC diagnosis and prognosis.

### LINC02159 exerts oncogenic roles in NSCLC progression

**Fig. 2:**
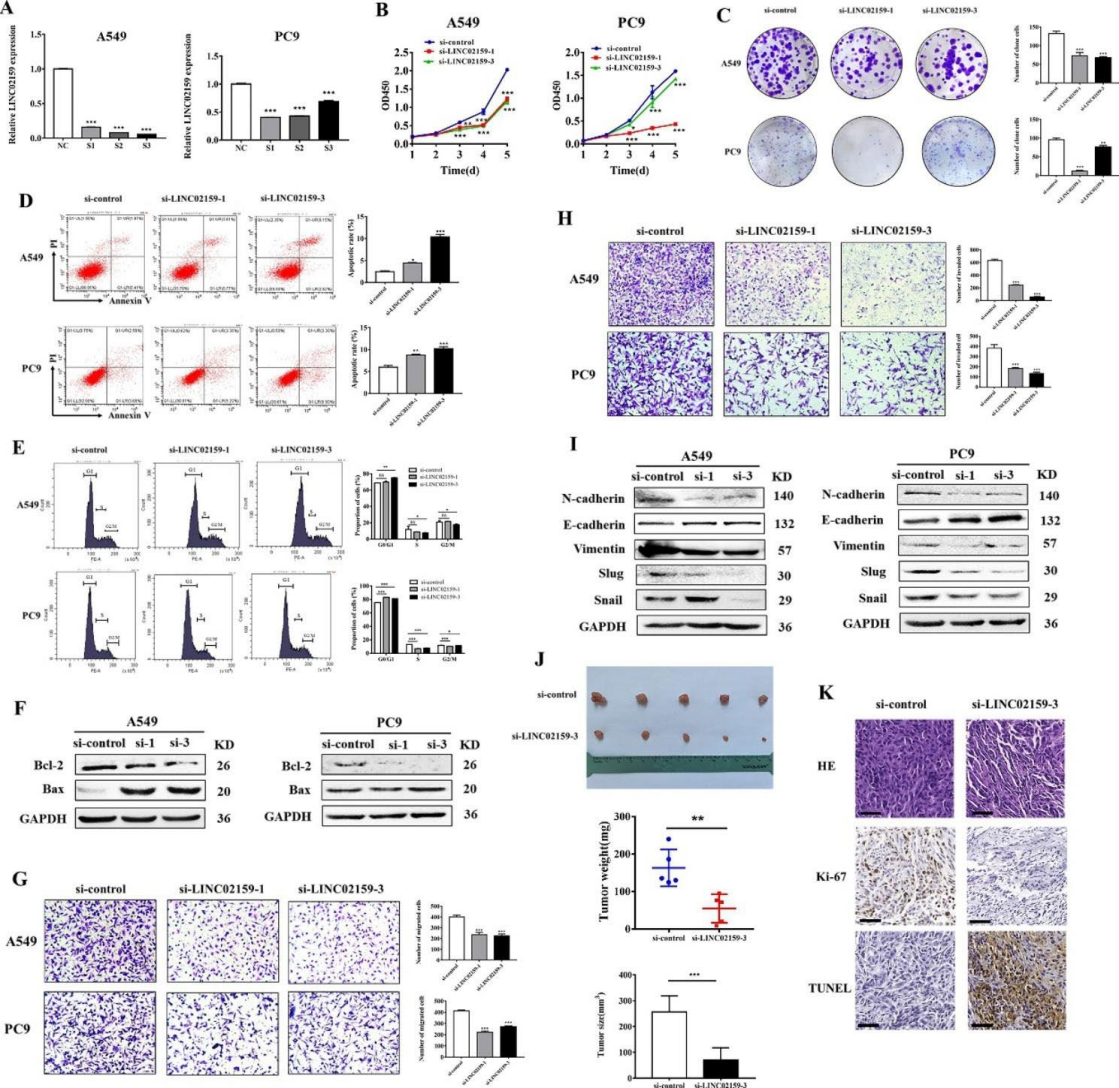
LINC02159 exerts oncogenic roles in NSCLC in vitro. **A** NSCLC cell lines A549 and PC9 cells were transfected with LINC02159 siRNA or control siRNA, and the knockdown efficiency was detected by qRT-PCR. **B** and **C** The proliferation capacity of A549 and PC9 cells after LINC02159 knockdown was evaluated by the CCK-8 assay (B) and colony-forming assay (C). **D** The percentage of apoptosis in the control group and LINC02159 knockdown group was analyzed by flow cytometry. **E** Flow cytometry analyses of cell cycle distribution after knockdown of LINC02159. Data are presented as mean ± SD (n = 3). **F** The effect of LINC02159 knockdown on the expression of proliferation and apoptosis-related proteins in A549 and PC9 cells. **G** Transwell migration assay was performed to determine the effects of LINC02159 knockdown on A549 and PC9 cell migration (200×). **H** The invasion ability of A549 and PC9 cells after LINC02159 knockdown was measured by using Matrigel invasion assay (200×). **I** Western blot analyses of EMT-related protein expression in control and LINC02159 knockdown groups. **J** The tumor weight and tumor size in mice of control and LINC02159 knockdown groups. **K** Sliced mouse tumor tissues were subjected to H&E, Ki-67, and TUNEL staining (scale bars = 100 μm). (*P < 0.05, **P < 0.01, ***P < 0.001, ns: no significance)

To reveal the biological functions of LINC02159 in NSCLC, we performed loss-of-function studies by using specific siRNAs against LINC02159 and verified the knockdown efficiency in NSCLC cells by qRT-PCR (Fig. 2A). The results of cell counting kit-8 (CCK-8) and cell colony formation assays showed that LINC02159 knockdown remarkably inhibited the proliferation of NSCLC cells (Fig. 2B and C). The results of flow cytometric analyses further proved that LINC02159 knockdown in NSCLC cells induced an increased number of apoptotic cells and cell cycle arrest at the G1 phase (Fig. 2D and E). In addition, the results of the western blot showed that LINC02159 knockdown upregulated the expression of Bax protein and downregulated that of Bcl-2 in NSCLC cells (Fig. 2F). Moreover, LINC02159 knockdown also suppressed the migrating and invading abilities of NSCLC cells (Fig. 2G and H). LINC02159 knockdown increased the expression of E-cadherin while decreasing that of N-cadherin and Vimentin as well as epithelial-mesenchymal transition (EMT) factors including Slug and Snail (Fig. 2I). To further validate the role of LINC02159 in NSCLC progression, we established in vivo mouse subcutaneous xenograft tumor models by using control and LINC02159 knockdown NSCLC cells. We found that mouse tumors in LINC02159 knockdown group had a smaller size and weight than those in the control group (Fig. 2J). The decreased number of proliferating tumor cells and increased number of apoptotic cells in mouse tumor tissues of the LINC02159 knockdown group were validated by IHC staining for Ki-67 and TUNEL staining (Fig. 2K). Furthermore, we overexpressed LINC02159 in NSCLC cells and found that it promoted the proliferation, migration, and invasion of NSCLC cells while inhibiting cell apoptosis and enhancing cell cycle progression (Figure S2). Subsequently, we evaluated the effect of LINC02159 knockdown on the sensitivity of NSCLC cells to chemotherapy, such as cisplatin (DDP) and 5-Fluorouracil (5-FU). The results showed that LINC02159 knockdown enhanced the sensitivity of NSCLC cells to DDP/5-FU treatment (Figure S3). In addition, we also found that LINC02159 knockdown decreased the IC_50_ values of gefitinib and erlotinib (Figure S3). Collectively, these results suggest that LINC02159 exerts oncogenic roles in NSCLC progression.

### LINC02159 interacts with ALYREF protein and positively correlates with ALYREF in NSCLC

**Fig. 3:**
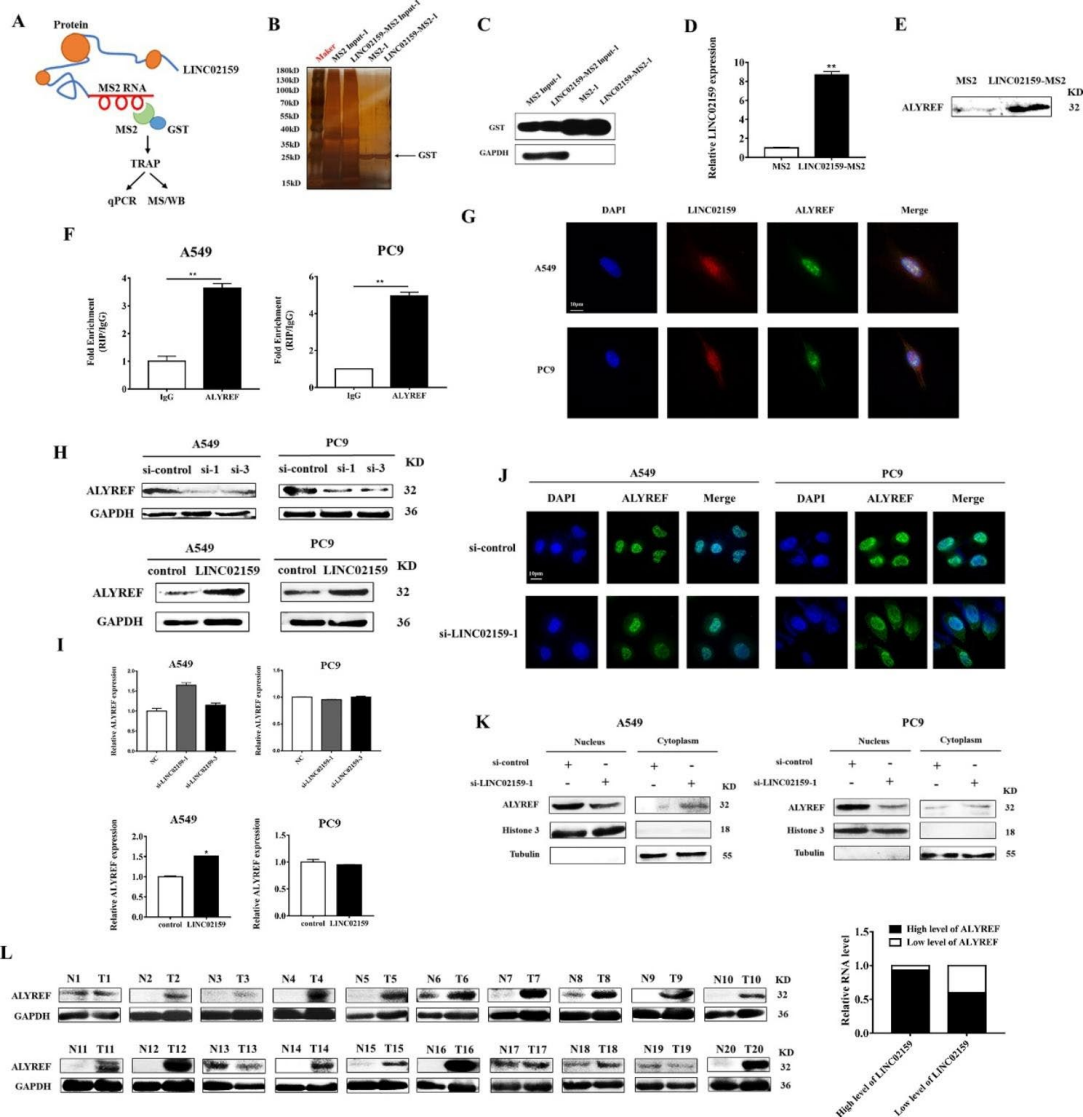
LINC02159 interacts with ALYREF protein and positively correlates with ALYREF in NSCLC. **A** Schematic diagram of the TRAP experiment. **B** and **C** Silver staining and western blot to detect the expression of GST after TRAP. **D** qRT-PCR was used to measure the expression level of LINC02159 in the product after TRAP. **E** LINC02159 was verified to bind to ALYREF by western blot. **F** RIP experiments for the interaction between ALYREF and LINC02159. **G** FISH and immunofluorescence staining for the co-localization of LINC02159 and ALYREF in NSCLC cells. **H** and **I** qRT-PCR and western blot analyses of ALYREF protein and mRNA expression in control and LINC02159-knockdown NSCLC cells. **J** Immunofluorescence staining of ALYREF in control and LINC02159-knockdown NSCLC cells. **K** Subcellular fractionation experiment for ALYREF protein in the nucleus and cytoplasm of control and LINC02159 knockdown NSCLC cells. **L** The expression of ALYREF protein in 20 paired NSCLC tissues and adjacent non-tumor tissues was detected by western blot. (*P < 0.05, **P < 0.01, ns: no significance)

To understand the mechanisms for the oncogenic roles of LINC02159 in NSCLC, we performed a TRAP assay to identify LINC02159-interacting proteins as it has been shown to mainly localize in the cell nucleus (Fig. 3A-D). Mass spectrometry results showed that several proteins were enriched in the LINC01259-MS2 group compared to the MS2 group. We chose ALYREF for the following study as it had the highest fold change. We verified the binding of LINC01259 and ALYREF by another independent TRAP assay (Fig. 3E). RIP assay results confirmed that LINC02159 was enriched in ALYREF-immunoprecipitated complex in NSCLC cells (Fig. 3F). Immunofluorescent staining results showed that LINC02159 co-localized with ALYREF in the nucleus of NSCLC cells (Fig. 3G). LINC02159 knockdown inhibited and overexpression enhanced the expression of ALYREF protein but had minimal effects on its mRNA (Fig. 3H and I). In addition, LINC02159 knockdown led to a re-localization of ALYREF protein from the nucleus to the cytoplasm (Fig. 3J and K). We then analyzed the expression of ALYREF protein in tumor and non-tumor tissues of NSCLC patients and found that ALYREF protein is highly expressed in 93% of tumor tissues that simultaneously harbored elevated levels of LINC02159 (Fig. 3L). In brief, these findings suggest that LINC02159 may perform tumor-promoting roles in NSCLC by interacting with ALYREF.

### ALYREF is upregulated in NSCLC and promotes NSCLC progression

**Fig. 4:**
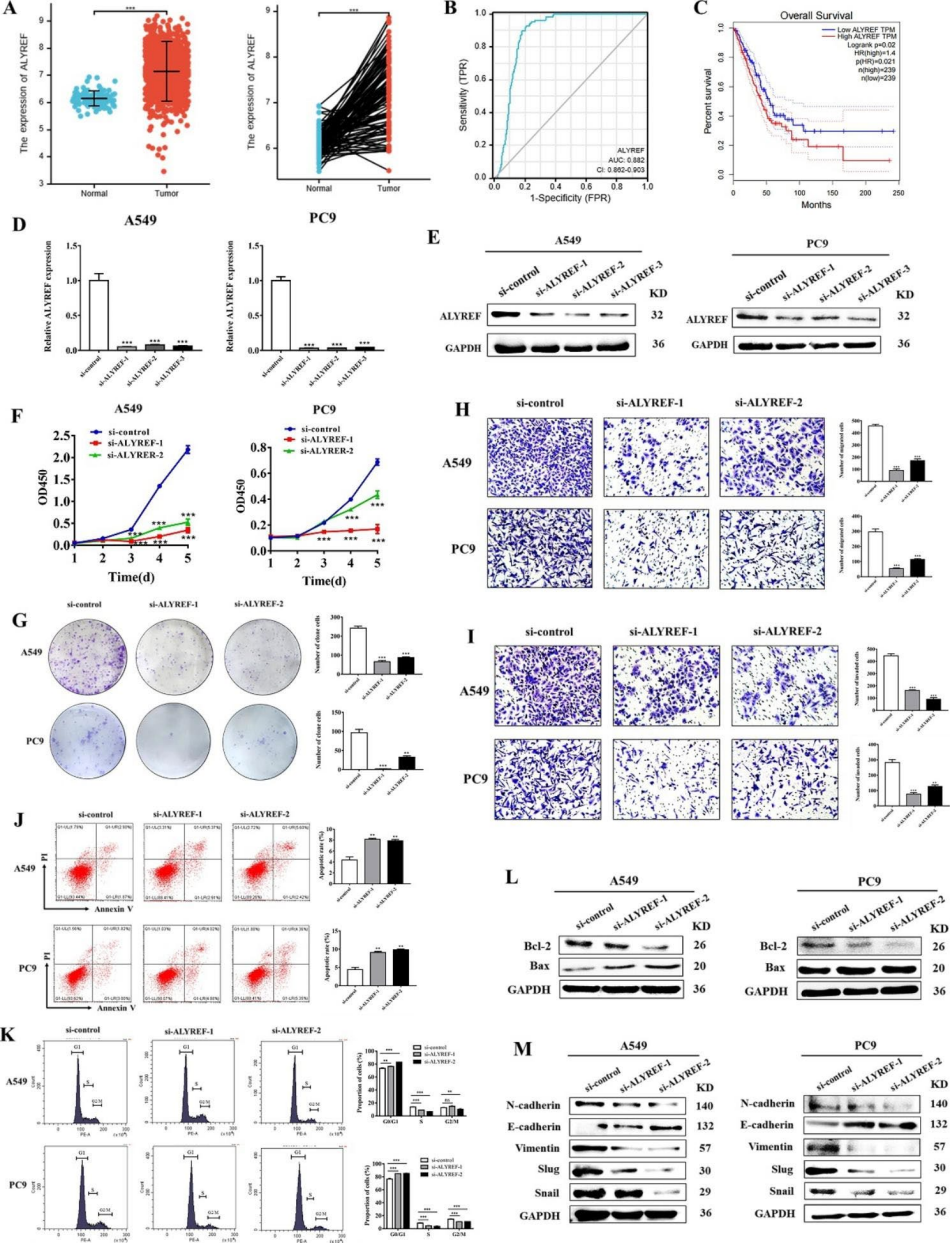
ALYREF is upregulated in NSCLC and promotes NSCLC cell proliferation, migration and invasion. **A** The expression of ALYREF in LUAD tissues (unpaired and paired samples) using data from TGCA. **B** ROC curve of ALYREF in NSCLC tissues from TCGA database. **C** Correlation analyses of ALYREF expression and NSCLC patients’ overall survival in the GEPIA database. **D** TCGA database analysis of the correlation between ALYREF expression and clinical stages. **E** and **F** Knockdown efficiency of ALYREF in A549 and PC9 cells was assessed by qRT-PCR and western blot. **G** Inhibited CCK-8 assays for the growth rate of control and ALYREF knockdown NSCLC cells. **H** The efficiency of plate colony formation in A549 and PC9 cells after ALYREF knockdown. **I** and **J** Transwell migration and Matrigel invasion assays for control and ALYREF knockdown groups (200×). **K** and **L** Flow cytometry analyses of cell apoptosis and cell cycle distribution in control and ALYREF knockdown groups. **M** and **N** Western blot were performed to determine the effects of ALYREF knockdown on the expression of growth and metastasis-related proteins in A549 and PC9 cells. (*P < 0.05, **P < 0.01, ***P < 0.001, ns: no significance)

ALYREF functions as a reader for m^5^C modification to regulate mRNA processing and nuclear export. Recent studies have shown that ALYREF is involved in tumor cell proliferation, metabolism, and metastasis [14–16]. The roles of ALYREF in NSCLC remain unclear although a recent study suggests that it, together with other m^5^C/m^6^A-related genes, could predict prognosis and immunotherapy efficacy in lung cancer [17]. Therefore, we first investigated the expression pattern and biological function of ALYREF in NSCLC. TCGA data analysis results showed that ALYREF was significantly upregulated in tumor tissues of NSCLC patients compared to non-tumor tissues, showing an AUC of 0.882 (Fig. 4A and B). Survival time analysis results showed that a high level of ALYREF predicted worse overall prognosis in NSCLC patients (Fig. 4C). Smilar to LINC02159, ALYREF also showed a tendency for upregulation in all-stage NSCLC patients (Figure S4). Loss-of-function studies further showed that ALYREF knockdown decreased NSCLC cell proliferation, migration, and invasion and induced cell apoptosis and cell cycle arrest (Fig. 4D-K). ALYREF knockdown upregulated the expression of Bax protein and downregulated that of Bcl-2 in NSCLC cells (Fig. 4L). ALYREF knockdown increased the expression of E-cadherin while decreasing that of N-cadherin and vimentin as well as EMT transcription factors including Slug and Snail (Fig. 4M). In addition, ALYREF knockdown not only improved the sensitivity of NSCLC cells to DDP/5-FU treatment, but also decreased the IC_50_ values of gefitinib and erlotinib (Figure S3). In summary, these results suggest that ALYREF is upregulated in NSCLC and acts as an oncogene to promote NSCLC progression.

### LINC02159 regulates YAP1 signaling in NSCLC through ALYREF-mediated m^5^C modification

**Fig. 5:**
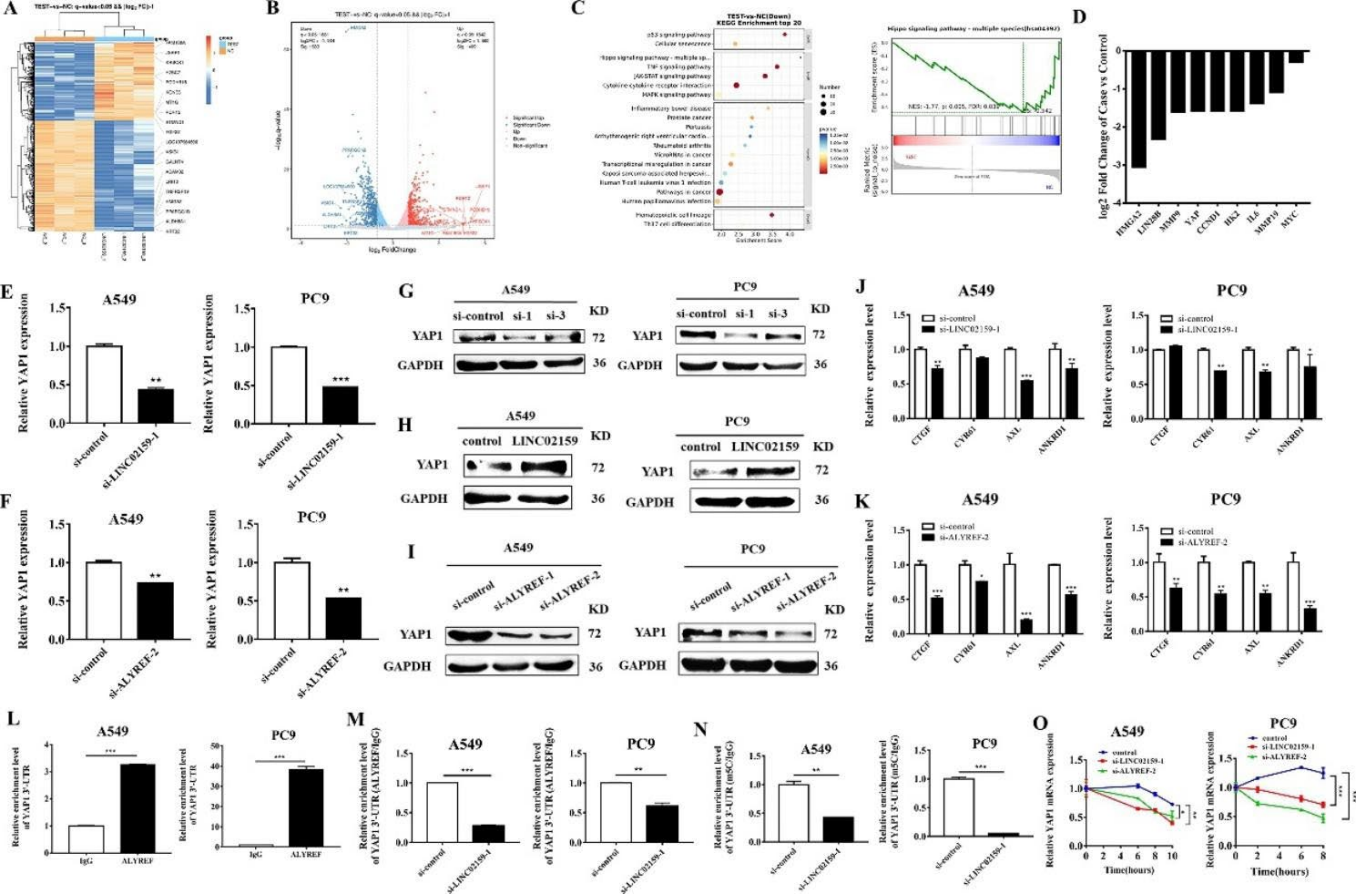
LINC02159 regulates YAP1 signaling in NSCLC through ALYREF-mediated m^5^C modification. **A** Hierarchical clustering showing differentially expressed mRNAs in control and LINC02159-knockdown groups. **B** Differential expression of mRNAs was displayed by the volcano map. **C** KEGG pathway enrichment analysis of down-regulated genes in RNA sequencing data. **D** The expression of tumor-related genes after LINC02159 knockdown according to RNA sequencing data. **E** YAP1 expression in NSCLC cells with LINC02159 knockdown. **F** YAP1 expression in NSCLC cells with ALYREF knockdown. **G**-**I** Western blot for YAP1 protein expression after knockdown (G) or overexpression (H) of LINC02159 and ALYREF knockdown (I). **J, K** The expression of Hippo pathway target genes after LINC02159 (J) and ALYREF knockdown (K). **L** RIP experiment for the interaction between ALYREF and 3’-UTR of YAP1. **M** RIP assays for the binding level of ALYREF to 3’-UTR of YAP1 after LINC02159 knockdown. **N** RIP assays for m^5^C level of the 3’-UTR of YAP1 in LINC02159-knockdown NSCLC cells. **O** The stability of YAP1 mRNA was measured in NSCLC cells with LINC02159 and ALYREF knockdown at the indicated time after Act D treatment (2 µg/mL). (*P < 0.05, **P < 0.01, ***P < 0.001, ns: no significance)

We next wanted to know the downstream gene and signaling pathway that is regulated by LINC02159 in NSCLC. RNA sequencing was performed to analyze the altered genes in LINC02159 knockdown NSCLC cells. The results showed that 409 genes were upregulated and 583 genes were downregulated in LINC02159 knockdown NSCLC cells compared to control cells (Fig. 5A and B). In addition, the KEGG enrichment analysis results showed that LINC02159 knockdown affected many pathways related to cancer, such as Hippo, JAK-STAT3, and MAPK signaling pathways (Fig. 5C). We chose 9 genes that were downregulated in LINC02159-knockdown NSCLC cells, including HMGA2, LIN28B, and YAP1, among others, as these genes have been demonstrated to be essential for cancer development and progression (Fig. 5D and S5). We focused on YAP1, as LINC02159 knockdown decreased its gene expression in NSCLC cells (Fig. 5E) and a recent study suggested that ALYREF may regulate YAP1 5-methylcytosine modification to increase its mRNA stability in lung cancer [18]. We confirmed that ALYREF knockdown decreased YAP1 gene expression in NSCLC cells (Fig. 5F). Western blot results showed that LINC02159 and ALYREF knockdown decreased while LINC02159 overexpression increased the expression of YAP1 protein (Fig. 5G-I). However, LINC02159 knockdown and overexpression had minimal effects on the expression of TAZ (Figure S6), indicating that the regulation of YAP1 by LINC02159 is selective. Previous studies have shown that YAP interacts with TEAD transcription factors to activate the expression of key target genes of the Hippo pathway, including CTGF, CYR61, AXL, and ANKRD1 [19]. Therefore, we detected the effects of LINC02159 and ALYREF knockdown on these target genes using qRT-PCR. The results showed that compared with the control group, the expression of Hippo pathway target genes was significantly decreased in LINC02159-, ALYREF- and YAP1-knockdown groups, indicating that LINC02159 regulates TEAD activity through YAP1 (Fig. 5J, K, and S7). We then determined the interaction of ALYREF and YAP1 mRNA by using a pair of primers specific to the 3’-UTR binding sites of ALYREF. RIP results showed that YAP1 mRNA was enriched in the ALYREF-immunoprecipitated complex in NSCLC cells (Fig. 5L). In addition, LINC02159 knockdown decreased the binding of ALYREF to YAP1 mRNA (Fig. 5M). Moreover, the m^5^C level of YAP1 3’-UTR was significantly decreased by LINC02159 knockdown (Fig. 5N). Subsequently, we showed that LINC02159/ALYREF knockdown weakened the stability of YAP1 mRNA using the Act D experiment (Fig. 5O). In summary, these results indicated that LINC02159 regulates the expression and stability of YAP1 mRNA by ALYREF-mediated m^5^C modification.

### LINC02159 promotes NSCLC progression through the YAP1/β-catenin axis

**Fig. 6:**
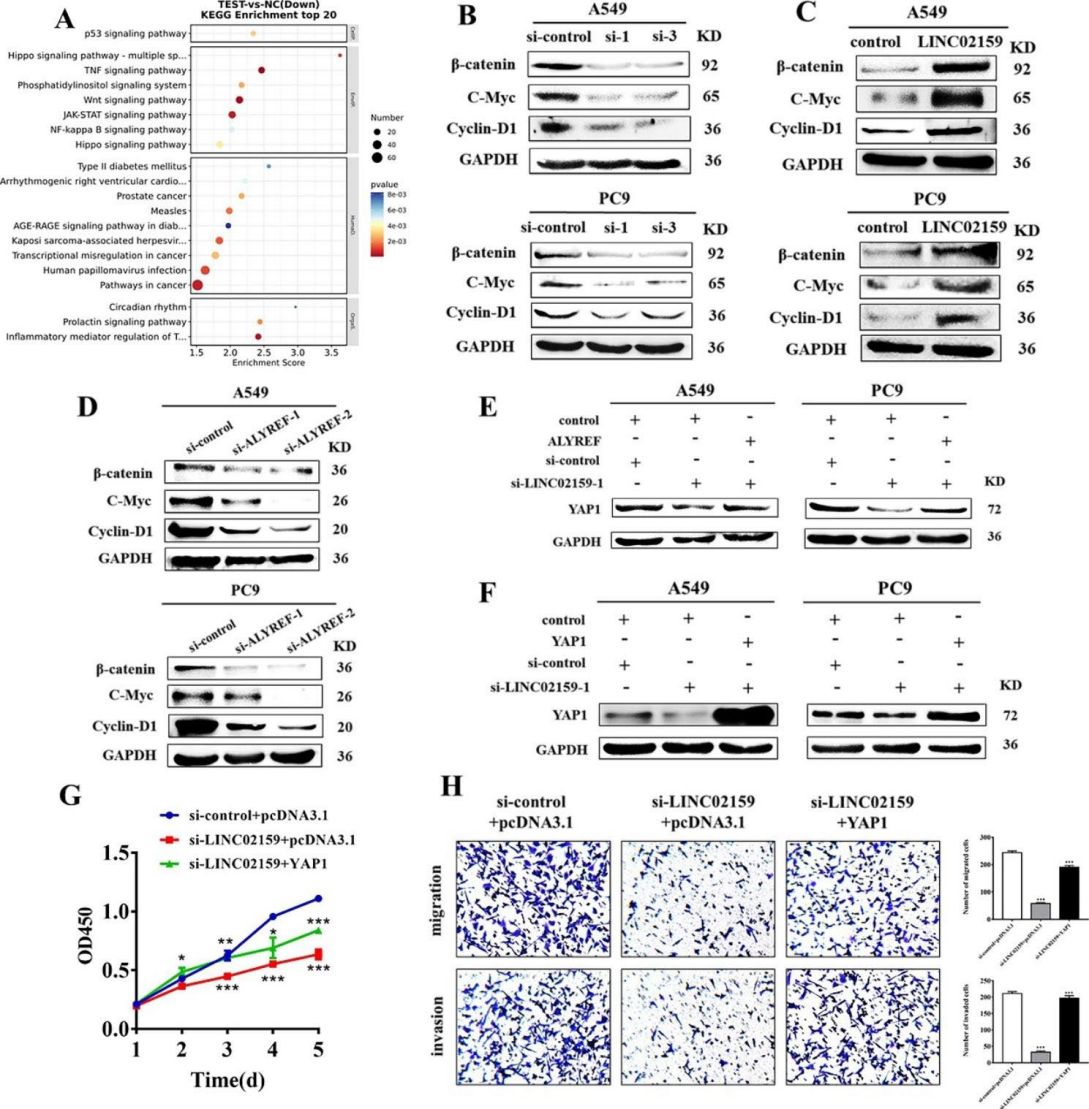
LINC02159 promotes NSCLC progression through the YAP1/β-catenin axis. **A** KEGG pathway enrichment analysis of down-regulated genes in RNA sequencing data (fold change > 1.5). **B-D** Western blot for the effects of LINC02159 knockdown/overexpression and ALYREF knockdown on the expression of Wnt/β-catenin pathway-related proteins. **E** Western blot for YAP1 protein expression in NSCLC cells with LINC02159 knockdown and ALYREF overexpression. **F** YAP1 protein expression in NSCLC cells with LINC02159 knockdown and YAP1 overexpression. **G** and **H** LINC02159 siRNA and YAP1 overexpression plasmids were co-transfected into PC9 cells. The proliferation (G), migration, and invasion (H) of PC9 cells were determined. (*P < 0.05, **P < 0.01, ***P < 0.001, 200×)

Previous studies indicated that YAP1 not only participates in the Hippo pathway as a transcriptional co-activator, but also as a key regulator of the Wnt/β-catenin signaling pathway. In addition, YAP1 can mediate the mutual crosstalk between them. In cancer, Wnt/β-catenin signaling persists on the “ON” state through gene mutation, while Hippo signaling is usually in the “OFF” state [20]. With *P* < 0.05 and fold change > 1.5 as the filter conditions, the KEGG enrichment analysis results showed that the Wnt/β-catenin signaling pathway was significantly enriched in LINC02159-knockdown group (Fig. 6A). Thus, we examined whether LINC02159 could also affect the activation status of the Wnt/β-catenin signaling pathway in NSCLC. Western blot results showed that, compared with the control group, the expression of β-catenin, c-Myc, and cyclin-D1 in LINC02159 and ALYREF-knockdown groups dramatically decreased, while the overexpression of LINC02159 led to an opposite effect (Fig. 6B-D). To further analyze the interaction of ALYREF, YAP1, and LINC02159, we knocked down LINC02159 and simultaneously overexpressed ALYREF in NSCLC cells. The data showed that overexpression of ALYREF could reverse the downregulation of YAP1 caused by the LINC02159 knockdown (Fig. 6E and S7). We then investigated whether the overexpression of YAP1 could reverse the effect of LINC02159 knockdown in NSCLC (Fig. 6F and S7). The results of gain- and loss-of-function experiments showed that knockdown of LINC02159 distinctly inhibited the expression of YAP1 and the growth, migration and invasion of NSCLC cells, but this inhibitory effect could be reversed by the overexpression of YAP1 (Fig. 6G-H). These results indicated that LINC01259 promotes NSCLC progression through the YAP1/β-catenin axis.

## Discussion

To further understand the roles of lncRNAs in NSCLC progression, we performed a transcriptomic analysis by using paired tumor tissues and adjacent normal tissues from NSCLC patients in this study. We identified a new lncRNA that has previously not been reported, LINC02159, which was upregulated in tumor tissues and serum of NSCLC patients. Intriguingly, the expression of LINC02159 was elevated in stage I NSCLC patients and maintained at a high level during all stages. The results of functional studies revealed that LINC02159 promoted NSCLC cell proliferation, migration, and invasion in vitro and *in vivo.* We further demonstrated that LINC02159 exerted tumor-promoting effect by binding to ALYREF, a regulator of RNA stability and gene expression. The interaction between LINC02159 and ALYREF improved YAP1 mRNA stability by enhancing its m^5^C modification, which increased its expression and activated the downstream Hippo and β-catenin pathways to promote NSCLC cell growth and metastasis. Our findings clarified the biological roles of LINC02159 and its mechanism of action in NSCLC progression, which may help us better understand the roles of LINC02159 in the pathogenesis of NSCLC and provides a promising biomarker and therapeutic target for NSCLC.

M^5^C is an important post-transcriptional RNA methylation modification. M^5^C methylation is controlled by m^5^C regulatory proteins, which includes methyltransferase as “writer” (DNMTs and NSUNs), de-methyltransferase as “eraser” (TETs), and RNA-binding proteins as “reader” (YTHDF2, ALYREF, and YBX1) [21]. Previously, Yang et al. have shown that m^5^C formation in mRNAs is mainly catalyzed by the RNA methyltransferase NSUN2, and m^5^C is specifically recognized by the mRNA export adaptor ALYREF [22]. RNA m^5^C methylation and its regulators (especially ALYREF) are significantly linked to many human diseases, including cancer [23]. The dysregulation of ALYREF has been reported in a variety of cancers. In neuroblastoma, MYCN amplification directly upregulates ALYREF transcription and ALYREF regulates MYCN stability in a forward feedback loop. ALYREF and MYCN form a transcriptional activator complex, which upregulates USP3 expression, promoting the growth and tumorigenicity of MYCN-amplified neuroblastoma cells [24]. Klec et al. show that a high level of ALYREF predicts poor survival in breast cancer patients. ALYREF activates the expression of lncRNA NEAT1 and selectively upregulates the short isoform at the post‑transcriptional level, which promotes breast cancer cell metabolism and progression [25]. Liu et al. have constructed a risk model by using 5 m^5^C RNA regulators, including ALYREF, to classify patients with lung cancer into high-risk (poorer OS) and low-risk (better response to immune checkpoint blockade therapy) groups [26]. Similarly, Ma et al. have constructed a prognostic risk model by using m^5^C/m^6^A-related gene signatures including ALYREF to evaluate prognosis and predict drug resistance and immunotherapy efficacy [17]. The functional roles of ALYREF in NSCLC have not been well studied thus far. Herein, we reported that ALYREF expression was upregulated in NSCLC tissues and its upregulation predicted poor survival in NSCLC patients. We further showed that ALYREF knockdown attenuated NSCLC cell proliferation, migration, and invasion, indicating that ALYREF plays oncogenic roles in NSCLC progression.

Several m^5^C-modified mRNAs have been identified as the targets of ALYREF in cancers, such as LRRC8A, Myc, PKM2, and YAP1, among others. Chen et al. demonstrated that LRRC8A is upregulated by NSUN2-mediated m^5^C modification and then m^5^C modified-LRRC8A mRNA is bound by YBX1 followed by increased RNA stability, which promotes cervical cancer progression through apoptosis suppression [27]. Wang et al. suggested that ALYREF is frequently increased in glioblastoma (GBM) tissues via MYC-mediated transcription. In turn, ALYREF drives GBM cell proliferation by activating the Wnt/β-catenin signaling pathway and stabilizing MYC mRNA [28]. Wang et al. demonstrated that, in bladder cancer cells, the elevation of ALYREF, activated by HIF-1α, stabilizes PKM2 mRNA via m^5^C modification and promotes bladder cancer cell proliferation by PKM2-mediated glycolysis [29]. In a recent study, Yu et al. demonstrated that m^5^C modification increases the stability of YAP1 mRNA and promotes the secretion of exosomes in lung cancer cells, which may be associated with their resistance to the treatment of third-generation EGFR-TKI [18]. Herein, we identified YAP1 as a downstream target of LINC02159 in NSCLC cells by integrating transcriptomic data and public databases and revealed that LINC02159 upregulated YAP1 expression by promoting ALYREF-mediated m^5^C modification and stabilizing YAP1 mRNA. Our findings provide additional evidence for the regulatory effect of m^5^C modification on YAP1 expression in cancer.

The regulation of m^5^C modification by lncRNA-mediated mechanism has not been well characterized. Recently, Yan et al. suggested that lncRNA FOXC2-AS1 is upregulated in gastric cancer (GC) tissues, and its high level is positively associated with advanced tumor node metastasis (TNM) stage and shorter OS in GC patients [30]. FOXC2-AS1 promotes the proliferation, migration, and invasion of GC cells by stabilizing FOXC2 mRNA. FOXC2-AS1 recruits RNA methyltransferase NSUN2 to FOXC2 mRNA, increasing its m^5^C level and repressing its degradation, indicating that FOXC2-AS1 exerts oncogenic roles in GC by regulating the stability of FOXC2 mRNA in an m^5^C-dependent manner. In this study, we reported that LINC02159 is bound to ALYREF to enhance its recognition of m^5^C-modified YAP1 mRNA, which stabilized YAP1 mRNA and increased its expression in NSCLC cells. Our findings support the notion that lncRNA may modulate RNA m^5^C modification by interacting with different m^5^C regulatory proteins. The detailed mechanism by which LINC02159 regulates the m^5^C modification of YAP1 mRNA by ALYREF deserves further investigation.

LncRNA-mediated Hippo/YAP1 pathway regulation has been reported in many cancers and has important roles in cancer progression [31]. There are versatile mechanisms for Hippo/YAP1 pathway regulation by lncRNAs in various cancers. For instance, Li et al. suggested that in pancreatic ductal adenocarcinoma cells, THAP9-AS1 regulates YAP1 through dual mechanisms. THAP9-AS1 acts as a competing endogenous RNA (ceRNA) for miR-484 to upregulate YAP1, and simultaneously binds to YAP1 protein to inhibit its phosphorylation-mediated inactivation by LATS1 [32]. Several other studies also demonstrated that lncRNAs may interact with YAP1 and inhibit or promote YAP1 phosphorylation and ubiquitination/degradation by LATS1 [33]. We showed in this study that LINC02159 upregulated YAP1 expression by stabilizing its mRNA through ALYREF-mediated m^5^C modification, which represents a new mechanism for the regulation of YAP1 in cancer.

In addition to serving as a co-factor for TEAD transcription factors in regulating Hippo signaling, YAP1 is also an important regulator of the Wnt/β-catenin pathway, whose dysregulation has been critically linked to cancer progression. For example, Kang et al. show that lncRNA SNHG3 and YAP1 are upregulated in laryngeal squamous cell carcinoma and SNHG3 binds to miR-340-5p to upregulate YAP1, thus activating the Wnt/β-catenin pathway to promote tumor progression [34]. Wang et al. suggested that GPNMB protein is highly expressed in diffuse large B-cell lymphoma, and activates the Wnt/β-catenin pathway by targeting YAP1 [35]. YAP1 is upregulated in NSCLC and its high level is negatively correlated with the survival time of NSCLC patients. The tumor suppressor KCTD11 is down-regulated in NSCLC and KCTD11 overexpression inhibits lung cancer progression by binding to β-catenin to regulate the activity of the Wnt and Hippo pathways [36]. We found here that LINC02159 upregulated YAP1 expression through ALYREF, which activated both Hippo and Wnt/β-catenin pathways, further proving the importance of YAP1 regulation by lncRNA in NSCLC progression.

**Fig. 7:**
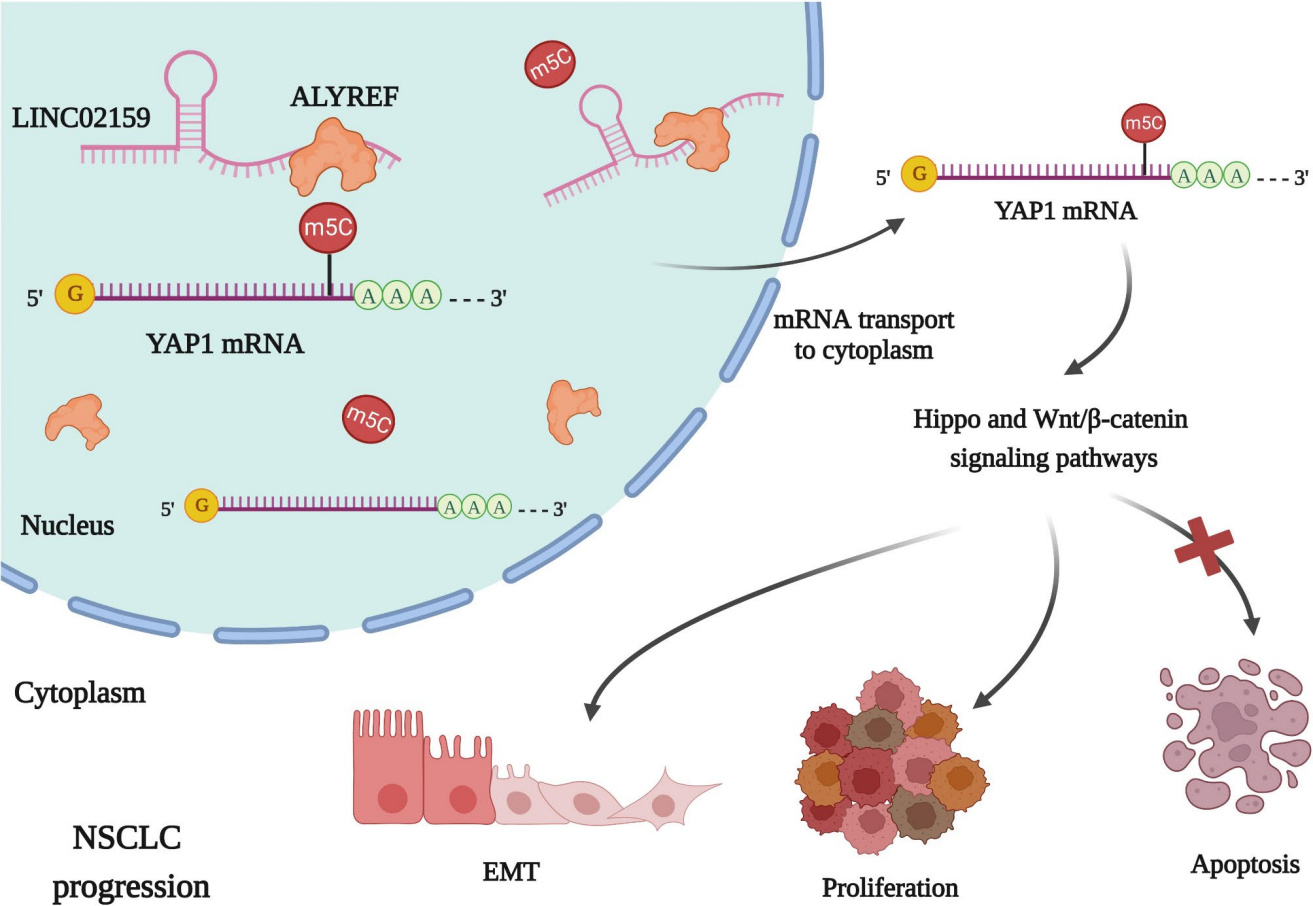
Proposed model for the biological role of LINC02159 in NSCLC progression. LINC02159 bounds to ALYREF to enhance the stability of YAP1 mRNA via m^5^C modification, leading to the overexpression of YAP1 and the activation of the Hippo and Wnt/β-catenin pathways, ultimately promoting the progression of NSCLC.

In conclusion, we showed for the first time, to the best of our knowledge, that a new lncRNA, LINC01259, promoted NSCLC progression by interacting with m^5^C modifier ALYREF to increase YAP1 m^5^C level and mRNA stability and expression (Fig. 7). LINC01259 and ALYREF were highly expressed and positively correlated in tumor tissues of NSCLC patients and high levels of ALYREF predicted an adverse outcome in NSCLC patients. Our study has uncovered the role of a new lncRNA in NSCLC progression and offers a potential diagnostic and prognostic biomarker and therapeutic target for NSCLC.

### Electronic supplementary material

Below is the link to the electronic supplementary material.

Supplementary Material 1

## Data Availability

All of the data and materials in this paper are available when requested.
