# Supplementary material for: LINC02159 promotes non-small cell lung cancer progression via ALYREF/YAP1 signaling
Source: Mol Cancer. 2023 Aug 4;22:122. doi: 10.1186/s12943-023-01814-x (PMC10401734; doi:10.1186/s12943-023-01814-x)

**Supplementary Materials and Methods**

**RNA extraction and quantitative real‑time PCR (qRT‑PCR)**

Total RNA was isolated from cells, tissue, and serum samples by Trizol (Vazyme, Nanjing, China) and reverse-transcribed into complementary DNA by HiScript 1st Strand cDNA Synthesis Kit (Vazyme) according to the manufacturer's procedure. qRT -PCR was conducted with SYBR Green Master Mix (Vazyme) and QuantStudio 3 system (Applied Biosystems, USA). The expression of U6 and/or β-actin was used as a control. Each experiment was repeated at least three times. Primer sequences are given in Additional file 1: Table S2.

**Protein extraction and western blotting**

Cells or tissues were lysed with RIPA buffer (Pierce, USA) containing mixed protease and phosphatase inhibitors. Protein concentrations were determined using the BCA kit (Vazyme). The extracted proteins were separated by sodium dodecyl sulfate-polyacrylamide gel electrophoresis and transferred to a 0.45 μm PVDF membrane (Roche, USA). Then, the membrane was blocked with 5% nonfat milk for 1-2 hours at room temperature. After blocking, the primary antibody was diluted with primary antibody dilution buffer (Beyotime, Shanghai, China) and incubated overnight at 4°C. After being washed three times, the membrane was incubated with the HRP-conjugated secondary antibody (Invitrogen) Protein levels were detected using a chemiluminescence gel imaging system (GE, USA). GAPDH was used as a control. All primary antibodies and their corresponding dilutions were listed in Additional file 1: Table S3.

**Cell proliferation assay**

Cells were placed in 96-well plates (1000-1500 cells/well) and cultured at different times. The optical density (OD) value at 450nm was detected by CCK-8 assay (Vazyme) every 24 hours.

**Colony formation assay**

Cells were seeded into 6-well plates (1000 cells/well) and cultured for 8-10 days. The medium was changed every 2–3 days. After being fixed with 4% paraformaldehyde (SCRC, China) and stained with crystal violet (Beyotime), the number of colonies in each group was counted.

**Transwell migration and Matrigel invasion assays**

Migration and invasion abilities were assessed using 24-Transwell migration chambers (8 μm pore) (Corning, USA). The transfected cells were collected and suspended in a serum-free medium. Cells (5-10×10^4^ for the migration assay and 10-20×10^4^ for the invasion assay) were plated into the upper chamber of an insert. The lower chamber was filled with a medium containing 10% fetal bovine serum. For the cell invasion assay, the upper chambers of the Transwell were pre-coated with diluted Matrigel (Corning, 1:4 dilution, 40 μL/well). After culturing for 24-48 hours, the transwell chambers were fixed with 4% paraformaldehyde and stained with crystal violet. Each well was counted and photographed.

**Cell cycle and apoptosis assay**

Cell cycle assay was conducted with a cell cycle detection kit (Fcmacs, China). The transfected cells were collected and fixed overnight in 95% ethanol and then stained with 50 μg/ml propidium iodide (PI) for 30 min in the dark. The percentage of cells in different phases was analyzed using flow cytometry (Merck Millipore, Germany). The Apoptosis Detection Kit (Fcmacs) was used to detect the cell apoptosis rate. At 24 hours after transfection, cells were harvested and resuspended in a binding buffer. Subsequently, cells were stained with Annexin V-Alexa Fluor 647 and PI and then incubated at room temperature for 15 min. Cell apoptosis rate was detected by flow cytometry.

**Subcellular fractionation**

Cytoplasmic and nuclear RNA was isolated using the Cytoplasmic & Nuclear RNA Purification kit (Norgen Biotek, Canada) according to the manufacturer's instructions, followed by reverse transcription and qRT-PCR. As for the separation of cytoplasmic and nuclear proteins, the Nuclear and Cytoplasmic Extraction Kit (Cwbio, China) was used and verified by western blot.

**Immunofluorescence**

Cell slides were washed twice with PBS, fixed with 4% paraformaldehyde, permeabilized with 0.1% Triton X-100, blocked with BSA for 1 hour, and then incubated with the anti-ALYREF antibody at 4°C overnight. After incubation with FITC-conjugated fluorescent secondary antibodies for 1 hour at room temperature, the cells were observed under a super-resolution microscope.

**Immunohistochemistry (IHC)**

The paraffin-embedded nude mouse tumor tissue sections were deparaffinized and rehydrated, incubated with primary antibodies against Ki-67 or stained with a TUNEL kit at 4°C overnight, and then incubated with secondary antibodies at room temperature for 30 min. After being incubated with diaminobenzidine substrate (DAB), the sections were counterstained with hematoxylin for microscopic examination.

**RNA stability assay**

The transfected cells were exposed to 2 μg/mL actinomycin D (Act D, Aladdin, China) at different times. Total RNAs were extracted at the indicated times and the level of YAP1 mRNA was detected by qRT-PCR.

**Bioinformatic analysis**

The expression levels and prognosis of LINC02159 and ALYREF genes were analyzed by GEPIA and TCGA-LUAD databases.

**Table S1: The sequences of siRNAs and probe**

| Name | Target sequence |
| --- | --- |
| si-Control | 5’-UUCUCCGAACGUGUCACGUTT  ACGUGACACGUUCGGAGAATT-3’ |
| si-LINC02159-1 | 5’-GGCCUGUGUAAACAUAACATT  UGUUAUGUUUACACAGGCCTT-3’ |
| si-LINC02159-2 | 5'-CAGCCCUGCACAUUAUGUATT  UACAUAAUGUGCAGGGCUGTT-3' |
| si-LINC02159-3 | 5’-CGCACUUAGAGAGAGUAAATT  UUUACUCUCUCUAAGUGCGTT-3’ |
| si-ALYREF-1 | 5’-GGAACAGCAGACGUGCACUTT  AGUGCACGUCUGCUGUUCCTT-3’ |
| si-ALYREF-2 | 5’-GCUUGUCACGUCACAGAUUTT  AAUCUGUGACGUGACAAGCTT-3’ |
| si-ALYREF-3 | 5’-GCGUAAACAGAGGUGGCAUTT  AUGCCACCUCUGUUUACGCTT-3’ |
| si-YAP1-1 | 5’-GCAUCUUCGACAGUCUUCUTT  AGAAGACUGUCGAAGAUGCTT-3’ |
| si-YAP1-2 | 5’-GACGACCAAUAGCUCAGAUTT  AUCUGAGCUAUUGGUCGUCTT-3’ |
| si-YAP1-3 | 5’-GGUGAUACUAUCAACCAAATT  UUUGGUUGAUAGUAUCACCTT-3’ |
| LINC02159  Homo probe | 5’-CATGGCAGTGGGCAGGAATG  CCACTGGGGACATAGAAAGC  AAAGCCGATGGGACAGGGAT-3’ |

**Table S2: Primer sequences for qRT-PCR**

| **Name** | **Primer** | **Sequence (5'-3')** |
| --- | --- | --- |
| β-actin | Forward | CACGAAACTACCTTCAACTCC |
|  | Reverse | CATACTCCTGCTTGCTGATC |
| U6 | Forward | CTCGCTTCGGCAGCACA |
|  | Reverse | AACGCTTCACGAATTTGCGT |
| LINC02159 | Forward | CCACCCCTTTCCCTGTAAGAG |
|  | Reverse | TTGGTCAAAGCCAAAAGCCG |
| ALYREF | Forward | CTATGATCGCTCTGGTCGCA |
|  | Reverse | ATGCCACCTCTGTTTACGCT |
| YAP1 | Forward | CCCTCGTTTTGCCATGAACC |
|  | Reverse | GCCTCTCCTTCTCCATCTGC |
| IL6 | Forward | CCACCGGGAACGAAAGAGAA |
|  | Reverse | GAGAAGGCAACTGGACCGAA |
| HK2 | Forward | GCCATCCTGCAACACTTAGGGCTTGAG |
|  | Reverse | GTGAGGATGTAGCTTGTAGAGGGTCCC |
| CCND1 | Forward | CCGAGAAGCTGTGCATCTAC |
|  | Reverse | CTTCACATCTGTGGCACAGAG |
| LIN28B | Forward | AGCAAAGGTGGTGGAGAAGA |
|  | Reverse | TCTCGGTTTATCATGGAGATG |
| ABCG4 | Forward | CCCCCTATTCCTTCAGTCCCC |
|  | Reverse | GACAGCTCCACGAACTCGAT |
| c-Myc | Forward | GGACTTGTTGCGGAAACGAC |
|  | Reverse | CTCAGCCAAGGTTGTGAGGT |
| MMP9 | Forward | TGTACCGCTATGGTTACACTCG |
|  | Reverse | GGCAGGGACAGTTGCTTCT |
| MMP19 | Forward | TGTACCGCTATGGTTACACTCG |
|  | Reverse | GGCAGGGACAGTTGCTTCT |
| TAZ | Forward | CCATCACTAATAATAGCTCAGATC |
|  | Reverse | GTGATTACAGCCAGGTTAGAAAG |
| CTGF | Forward | CCAATGACAACGCCTCCT |
|  | Reverse | TGGTGCAGCCAGAAAGCTC |
| CYR61 | Forward | AGCCTCGCATCCTATACAACC |
|  | Reverse | TTCTTTCACAAGGCGGCACTC |
| AXL | Forward | GGAGCCCAACAACTTCTGAGG |
|  | Reverse | GGACTTTCTTCAGCCTGCGTG |
| ANKRD1 | Forward | CACTTCTAGCCCACCCTGTGA |
|  | Reverse | CCACAGGTTCCGTAATGATTT |

**Table S3: Antibodies used in this study**

| Antigens | Manufacturers | Applications | |
| --- | --- | --- | --- |
| E-cadherin | Cell Signaling Technology, Beverly, MA, USA | | 1:1000 for WB |
| N-cadherin | Cell Signaling Technology, Beverly, MA, USA | | 1:1000 for WB |
| β-catenin | Cell Signaling Technology, Beverly, MA, USA | | 1:1000 for WB |
| Vimentin | Cell Signaling Technology, Beverly, MA, USA | | 1:1000 for WB |
| Slug | Cell Signaling Technology, Beverly, MA, USA | | 1:1000 for WB |
| c-Myc | Cell Signaling Technology, Beverly, MA, USA | | 1:1000 for WB |
| cyclin D1 | Cell Signaling Technology, Beverly, MA, USA | | 1:1000 for WB |
| GAPDH | Cell Signaling Technology, Beverly, MA, USA | | 1:2000 for WB |
| Snail | Proteintech, Chicago, USA | | 1:500 for WB |
| Bcl-2 | Proteintech, Chicago, USA | | 1:500 for WB |
| Bax | Proteintech, Chicago, USA | | 1:1000 for WB |
| ALYREF | Abcam, Cambridge, MA, USA | | 1:2000 for WB,  5μg/T for IP |
| YAP1 | Abcam, Cambridge, MA, USA | | 1:1000 for WB |
| m^5^C | Abcam, Cambridge, MA, USA | | 5μg/T for IP |
| Ki-67 | Cell Signaling Technology, Beverly, MA, USA | | 1:200 for IHC |
| TUNEL | Cell Signaling Technology, Beverly, MA, USA | | 1:200 for IHC |

**Supplementary Figure Legends**

**Fig S1** The correlation between CEA and LINC02159 expression and TCGA database analysis for LINC02159 expression in NSCLC. **A** The correlation between CEA and LINC02159 expression in the serum of NSCLC patients. **B** TCGA database analysis of the correlation between LINC02159 expression and clinical stage. **C** The correlation between LINC02159 expression and the survival time of NSCLC patients according to the TCGA database. (****P*<0.001).


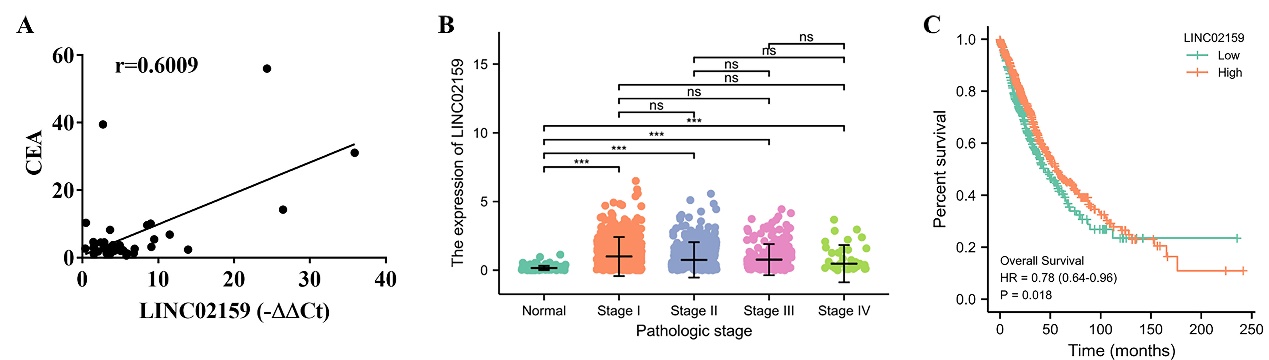


**Fig S2** Overexpression of LINC02159 promotes NSCLC cell proliferation, migration and invasion in vitro. **A** The efficiency of LINC02159 overexpression in NSCLC cells was examined by qRT-PCR. **B** The proliferation of control and LINC02159 overexpression NSCLC cells was measured by CCK-8 assays. **C** The impact of LINC02159 overexpression on NSCLC cell proliferation was evaluated by cell colony formation assay. **D** and **E** The migration and invasion abilities of control and LINC02159-overexpressing NSCLC cells were determined by Transwell migration and Matrigel invasion assays. **F** and **G** Cell apoptosis and cycle distribution were determined by flow cytometry. Data are shown as mean ± SD (n=3). **H** and **I** Western blot was performed to determine the effects of LINC02159 overexpression on the expression of growth and metastasis-related proteins in NSCLC cells. (**P*<0.05, ***P*<0.01, ****P*<0.001, ns: no significance).


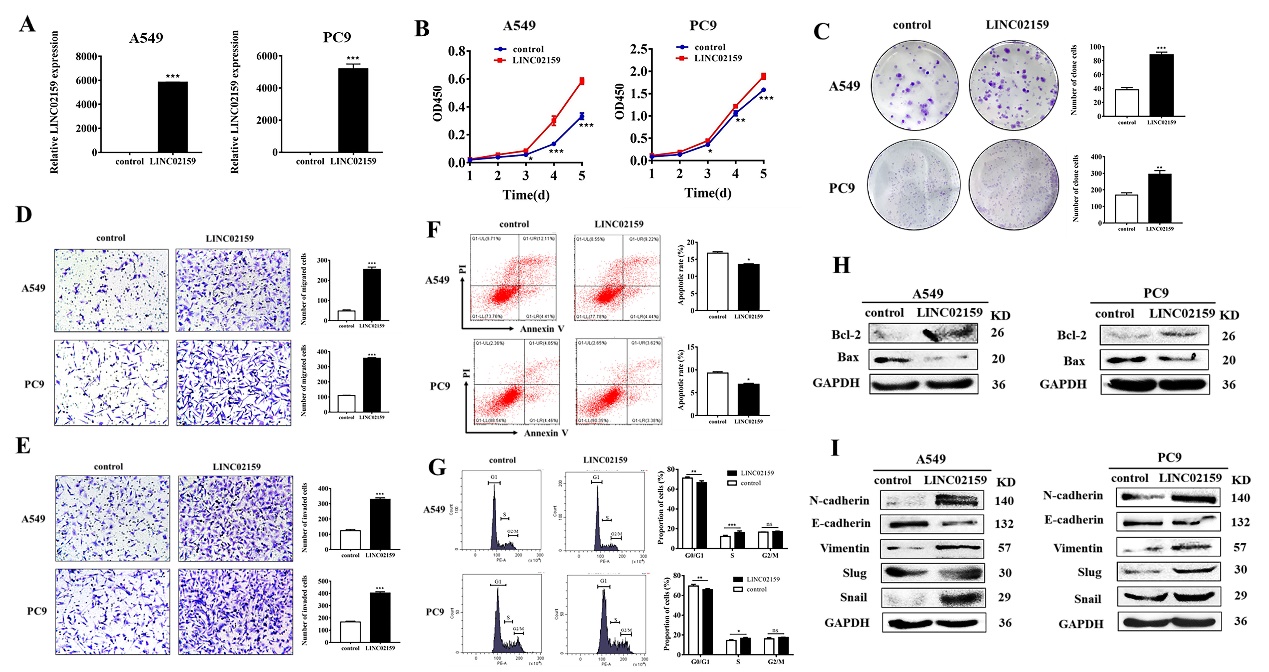


**Fig S3** The effects of LINC02159 and ALYREF knockdown on the sensitivity of NSCLC cells to chemotherapy. **A** The effects of LINC02159 and ALYREF knockdown on the sensitivity of NSCLC cells to DDP treatment. **B** The effects of LINC02159 and ALYREF knockdown on the sensitivity of NSCLC cells to 5-FU treatment. **C** The effects of LINC02159 and ALYREF knockdown on the sensitivity of NSCLC cells to gefitinib treatment. **D** The effects of LINC02159 and ALYREF knockdown on the sensitivity of NSCLC cells to erlotinib treatment.


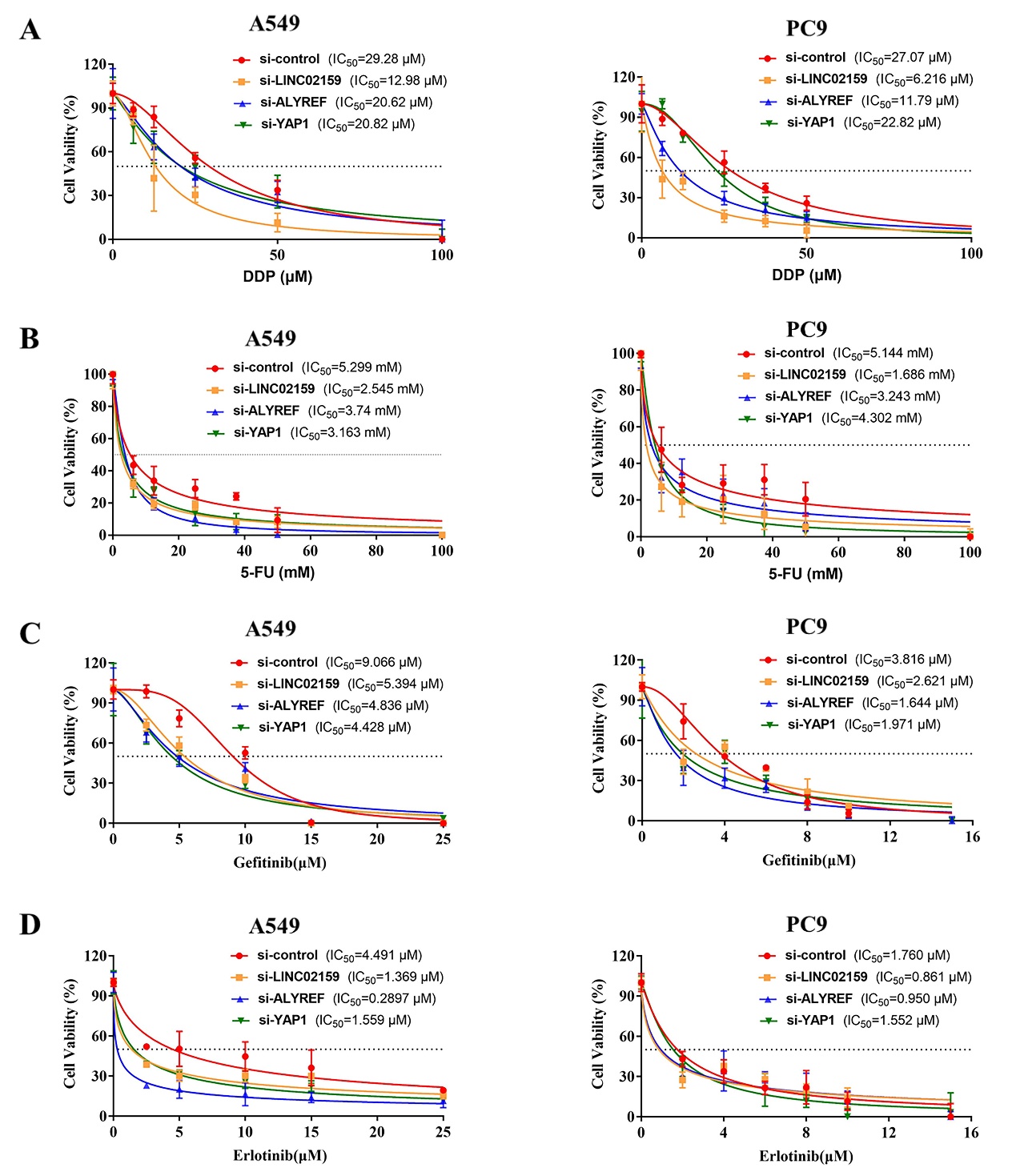


**Fig S4** The correlation between ALYREF expression and clinical stages of NSCLC according to the TCGA database.


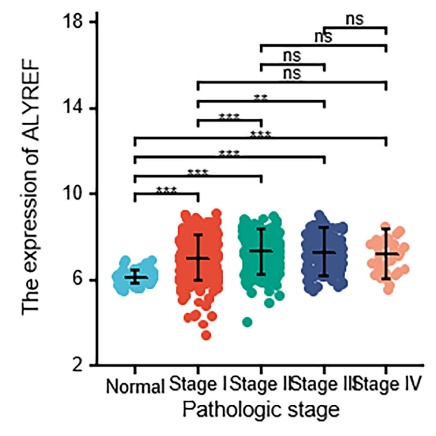


**Fig S5** Screening of LINC02159 downstream target genes. **A** The expression of differential genes in NSCLC cells after LINC02159 knockdown. **B** The expression of differential genes in NSCLC cells after LINC02159 overexpression. **C** The expression of differential genes in NSCLC cells after ALYREF knockdown. (****P*<0.001, ***P*<0.01, **P*<0.05, ns: no significance).


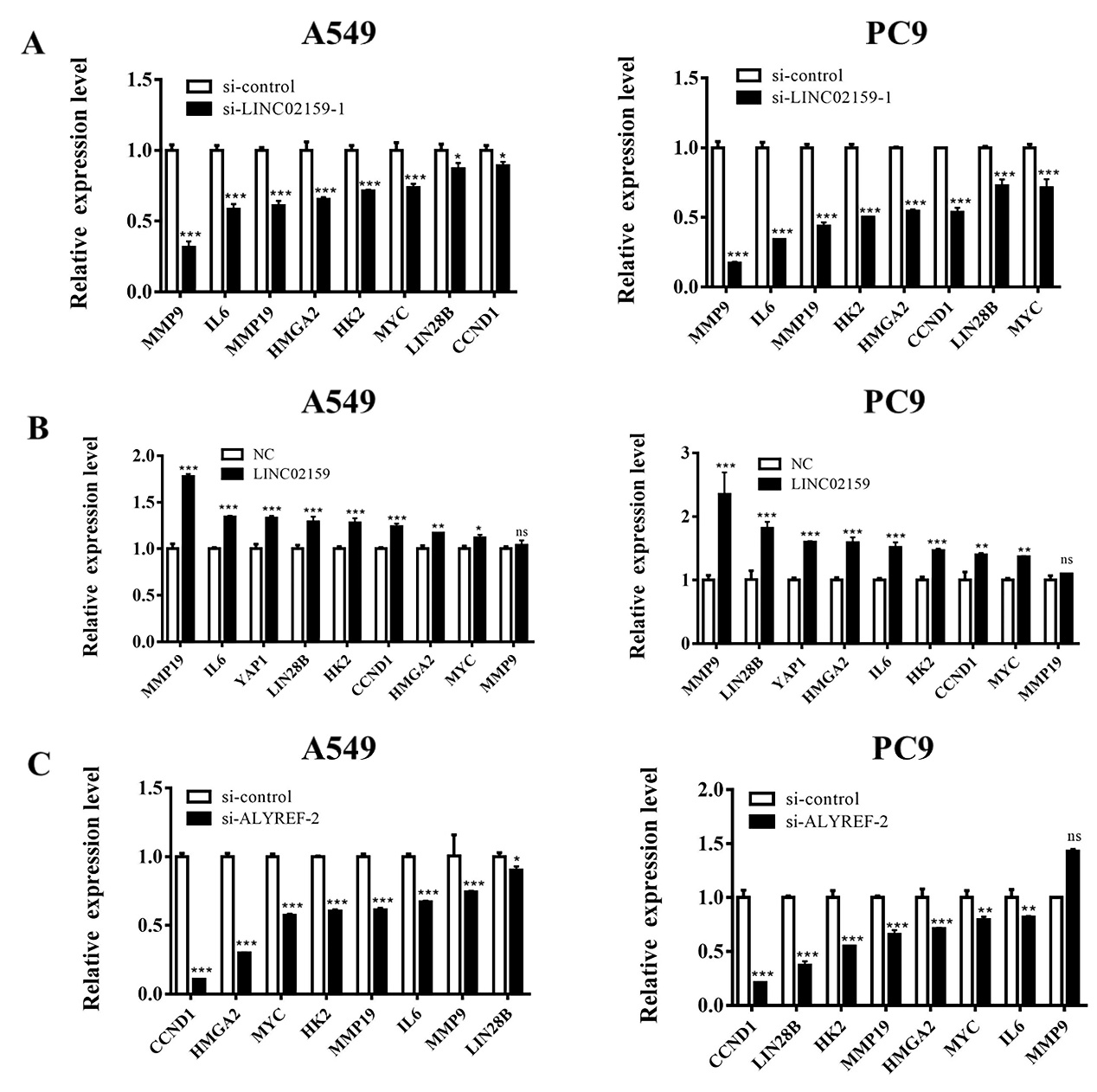


**Fig S6** The effects of LINC02159 knockdown and overexpression on TAZ expression. (ns: no significance).


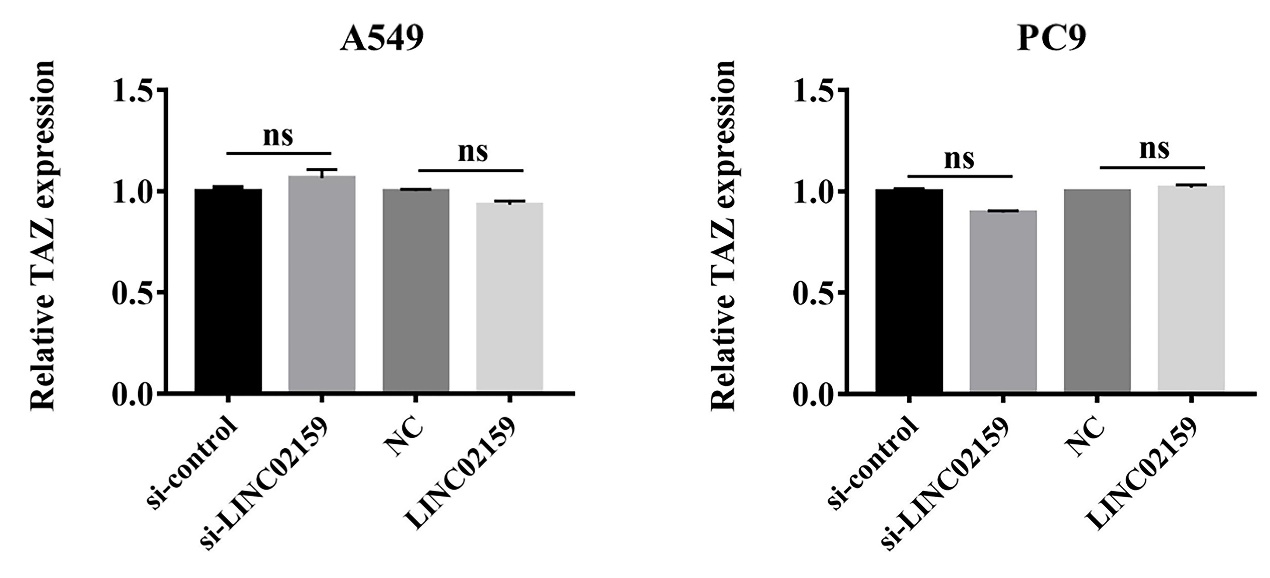


**Fig S7** The expression of Hippo pathway target genes after YAP1 knockdown. (****P*<0.001, ***P*<0.01, **P*<0.05).


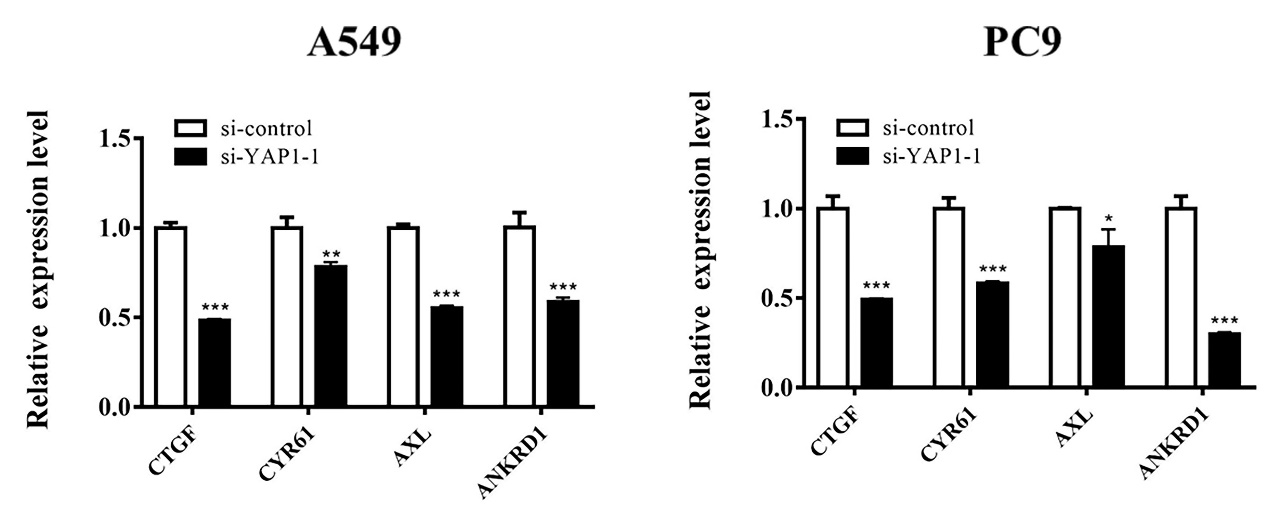


**Fig S8** The effects of ALYREF/YAP1 overexpression on the role of LINC02159 knockdown in NSCLC. **A** qRT-PCR analysis of YAP1 gene expression in NSCLC cells with LINC02159 knockdown and ALYREF overexpression. **B** qRT-PCR analyses of YAP1 gene expression in NSCLC cells with LINC02159 knockdown and YAP1 overexpression. (**P*<0.05, ***P*<0.01, ****P*<0.001).


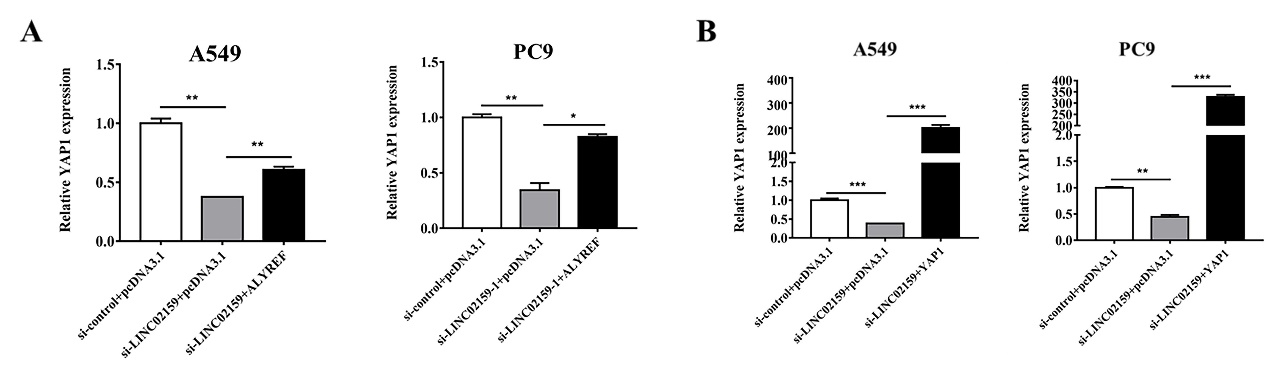

Supplement: Supplementary file 1 — Supplementary Material 1 [file 12943_2023_1814_MOESM1_ESM.docx]
